# Silica Containing Composite Anion Exchange Membranes by Sol–Gel Synthesis: A Short Review

**DOI:** 10.3390/polym13111874

**Published:** 2021-06-04

**Authors:** Emanuela Sgreccia, Riccardo Narducci, Philippe Knauth, Maria Luisa Di Vona

**Affiliations:** 1 Department of Industrial Engineering and International Laboratory “Ionomer Materials for Energy”, University of Rome Tor Vergata, I-00133 Rome, Italy; riccardo.narducci@uniroma2.it (R.N.); divona@uniroma2.it (M.L.D.V.); 2 CNRS, Madirel (UMR 7246) and International Laboratory “Ionomer Materials for Energy”, Aix Marseille University, F-13013 Marseille, France; philippe.knauth@univ-amu.fr

**Keywords:** ionomers, ormosils, diffusion dialysis, fuel cells, redox flow batteries, electrodialysis

## Abstract

This short review summarizes the literature on composite anion exchange membranes (AEM) containing an organo-silica network formed by sol–gel chemistry. The article covers AEM for diffusion dialysis (DD), for electrochemical energy technologies including fuel cells and redox flow batteries, and for electrodialysis. By applying a vast variety of organically modified silica compounds (ORMOSIL), many composite AEM reported in the last 15 years are based on poly (vinylalcohol) (PVA) or poly (2,6-dimethyl-1,4-phenylene oxide) (PPO) used as polymer matrix. The most stringent requirements are high permselectivity and water flux for DD membranes, while high ionic conductivity is essential for electrochemical applications. Furthermore, the alkaline stability of AEM for fuel cell applications remains a challenging problem that is not yet solved. Possible future topics of investigation on composite AEM containing an organo-silica network are also discussed.

## 1. Introduction

Anion exchange membranes (AEM) are important materials for applications in energy and in the environment [1,2,3,4]. In electrochemical energy technologies, they are used as ion-conducting separators between the electrode compartments physically impeding the mixture of electrolyte solutions in redox flow batteries [5,6,7,8,9,10] or gases (hydrogen and oxygen) in anion exchange membrane fuel cells (AEMFC) and water electrolyzers [11,12,13,14]. In this case, the major requirements are a high ionic conductivity in order to reduce as much as possible the Ohmic drop during current flow; and a low permeability to reactants, i.e., electrochemically active ions in a redox flow battery or a low hydrogen and oxygen permeability in AEMFC. The environmental applications of AEM are especially for water purification [15,16] or acid recovery by diffusion dialysis (DD) [17,18]. In this case, the ion permselectivity is a major factor of merit [19,20]. In any case, AEM must present a good chemical stability against acids and bases; reducing or oxidizing conditions; a good mechanical stability, especially high strength and sufficient ductility; and a good thermal stability to be applicable above room temperature, typically up to 100 °C [21,22,23].

The field of advanced membrane separators can profit from two approaches: (i) the synthesis of new ionomers with new polymer backbones or new ion exchange groups; and (ii) the modification of existing ionomers, especially by adding a second phase to prepare composite ion exchange membranes.

We have contributed a significant amount of work, along with others, on the synthesis and characterization of composite ion exchange membranes either in protonic or anionic forms. Most of the published literature concern materials with inorganic second particles, especially silica [24,25,26,27,28]; titania [29,30,31,32] or more complex inorganic solids, such as MXenes [33]; or those presenting an intrinsic ionic conductivity, such as the proton-conducting Zr(HPO_4_)_2_ [34,35] or anion-conducting lamellar double hydroxides (LDH) [36,37,38,39].

In most of the cases, preformed oxide particles were added to the anion exchange polymer during casting. This procedure can, however, lead to an imperfect distribution of the nanoparticles due to sedimentation. This phenomenon generally impacts mechanical properties with a lower strength and ductility due to the inhomogeneities and lower performances, especially reduced ionic conductivity. An interesting approach to avoid this drawback is the in situ formation of the second phase by sol–gel chemistry inside the casting solution.

The sol–gel process that generates in situ inorganic or hybrid networks within a polymeric membrane is a flexible and versatile strategy for the synthesis of conductive nanocomposite materials with very homogeneous and precise morphology [40,41,42]. In principle, the sol–gel process begins with the infiltration of a precursor solution into the polymer matrix. The hydrolysis of the precursor occurs due to the nucleophilic water present in the membrane that reacts with the inorganic atoms. If the reaction is acid-catalyzed a hydrated acidic polymer, such as Nafion, acts as a catalyst by itself. The original morphology of unfilled polymer membranes is maintained even after the sol–gel process. The differences between anionic and cationic membranes are considerable and synthetic methods for preparing nanocomposites via sol–gel chemistry cannot be fully transposed between the two ionic conductors but must be targeted to the chosen material.

Many silica precursors are available. An important role in the formation of a hybrid silica network is played by organically modified silicas (ORMOSIL), where the organic functional groups are covalently attached to the silica structure which consents to a specific control of the chemical and physical properties of the materials [42,43]. The number of alkoxide groups on the silicon atom determines the role in the reaction and the network structure: tetra-functional silicon alkoxides act as a network former, tri-functionals behave as cross-linker, di-functionals are bridging molecules, and mono-functionals can be used as terminating agents.

This short review is intended to present a comprehensive view on anion-conducting hybrid polymers obtained by sol–gel routes from silica precursors covering the last 15 years. It is subdivided into three parts corresponding to the major applications.

## 2. Membranes for Diffusion Dialysis (DD) and Related Fields

AEM are a key component that determines the performance of DD processes, especially for acid recovery. Pioneers in this field were Tongwen Xu and his co-workers that, starting from 2003, prepared various hybrid materials in order to test the feasibility of membranes containing a silica network as anion exchangers. Initially they prepared positively charged co-polymers and started from polyethylene oxide (PEO) functionalized with alkoxysilanes followed by quaternization [44]. The authors also explored aliphatic co-polymers formed by glycidylmethacrylate (GMA) and 3-methacryloxy propyltrimethoxysilane (MPS) [45].

Glycidyl (or 2,3-epoxypropyl) groups are often introduced in polymeric backbones due to their high reactivity which allows easy polymer functionalization using various reagents such as amine, water, alcohol, alkyl halide, etc. [46]. The co-polymers formed by a polymerizable unit containing a glycidyl moiety and an ORMOSIL in the backbone were modified by ring opening reactions and used as the precursor for sol–gel processes with further MPS [45].

**Figure 1:**
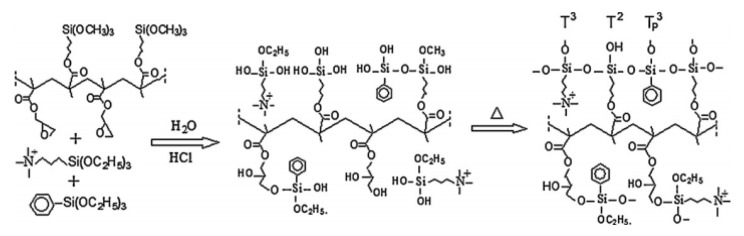
Schematic synthesis of AEM showing the role of glycidyl and alkoxysilane groups. Reprinted from Reference [47] with permission from Elsevier.

The same strategy was applied to the co-polymer formed by GMA and MPS and reacted by sol–gel with N-triethoxysilylpropyl-N,N,N-trimethylammonium iodide (APTEOS-I) and monophenyltriethoxysilane (EPh) (Figure 1). The homogeneity was strongly influenced by both the quantity of silicon and the molecular weight of the copolymer: membranes with higher molecular weight showed the worst morphological properties [47]. A related approach used MPS, 3-glycidoxypropyltrimethoxysilane (GPTMOS), and triethoxysilylpropylamine quaternized with CH_3_I to introduce the anion-exchange moiety [48].

Some authors started from a commercial aromatic polymer, mostly poly (2, 6-dimethyl-1,4-phenylene oxide) (PPO) and post-functionalized the backbone with an ORMOSIL, often by methylbromination or methylchlorination routes, to achieve a hybrid precursor for the following sol–gel processes.

For example, Xu et al. functionalized brominated PPO (BrPPO) with 3-aminopropyl-trimethoxysilane (APTMOS) followed by sol–gel reaction with further APTMOS [49]. The material became an anionic conductor during the sol–gel procedure in acidic media due to the protonation of the amine moieties. Throughout this explorative work, the authors showed that a good homogeneity can be achieved controlling the initial ratio between APTMOS and the degree of bromination of PPO. Hollow-fibers of BrPPO, obtained by electrospinning, were treated with tetraethoxysilane (TEOS) and then quaternized [50]. The membranes presented a homogenous morphology and good thermal and dimensional stabilities.

Analogous membranes that were based on PPO and were reacted with EPh and TEOS were applied in DD [51]. The operational temperature was in the range from 15 °C to 65 °C and the separation performances increased at higher temperatures. This material was also electrospun and hot-pressed. The resulting membranes showed excellent DD characteristics attributed to the particular membrane morphology [52].

**Figure 2:**
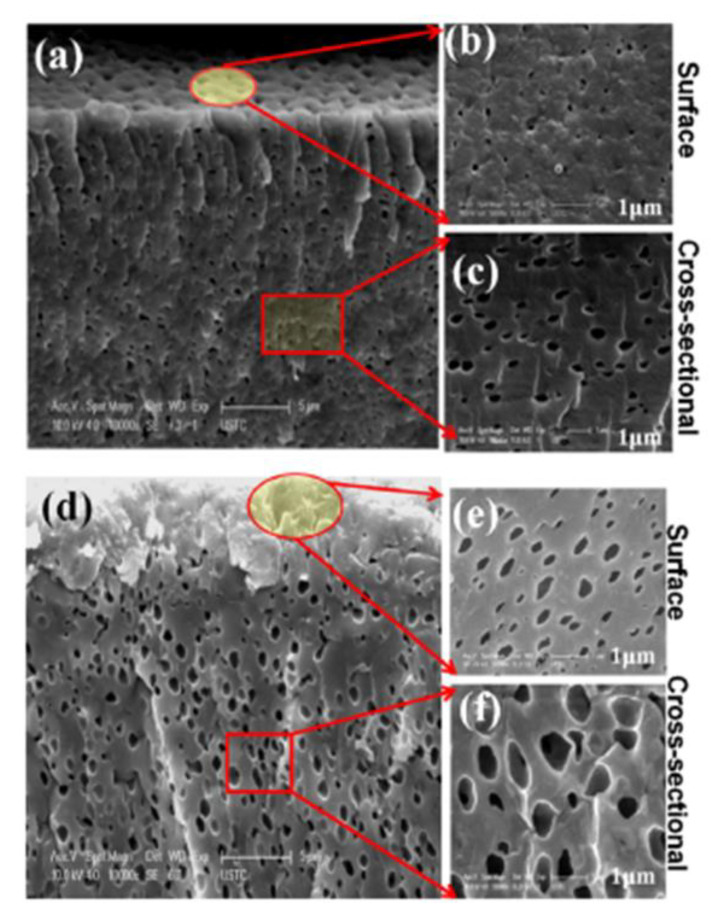
Scanning electron micrographs of porous AEM with ionic liquid: (**a**–**c**) corresponds to a low amount of ionic liquid, while (**d**–**f**) has a higher ionic liquid loading. Reprinted from Reference [53] with permission from Elsevier.

PPO quaternized with dimethylaminoethanol (DMAE) was used for the sol–gel reaction with the ionic liquid 3-methyl-1-(3-(triethoxysilyl) propyl)-1H-imidazolium chloride [53]. The interest in this work is to maintain the ion exchange capacity by adding a silica network containing ionic groups. The obtained membranes were thin, porous, mechanically stable, and possessed a high ion exchange capacity above 2.1 meq/g and an excellent performance in DD. The porosity increases with the amount of ionic liquid (Figure 2).

In an extension of this work, amphoteric membranes were synthetized by adding the same ionic liquid and 4-(hydroxymethyl) benzoic acid to PPO quaternized with DMAE [54]. The zwitterionic pores not only improved the proton diffusion coefficients but also the diffusion of ferrous ions (feed solution HCl and FeCl_2_) showing that the size of the pores was of paramount importance to achieve high selectivity.

Another approach used poly (vinyl alcohol) (PVA) as the backbone. Major advantages of PVA are its high hydrophilicity that improves water diffusion, its non-toxicity, and its low price. Furthermore, the hydroxyl groups can easily react by condensation with organo-silica precursors. Many efforts were spent to improve the mechanical properties of PVA.

Xu and his co-workers prepared hybrid materials based on poly (vinyl alcohol) (PVA) and APTEOS-I [55]. In order to improve the mechanical properties, the PVA matrix was cross-linked with different agents ranging from small alkoxysilanes (TEOS, GPTMOS, and EPh) to the co-polymer formed by GMA and MPS, which is described in Reference [47]. Membranes obtained with the co-polymer cross-linker showed the best properties in DD tests.

Hybrid membranes were prepared from TMA-quaternized PPO, PVA, and double crosslinking agents including EPh and TEOS [56]. The OH groups in PVA were regarded as assistant functional groups for the sorption and DD process. Similar membranes were later used for acid recovery [57]. Hybrid quaternized PVA (Q-PVA) based membranes were also used for the pervaporation dehydration of ethanol [58]. Q-PVA was obtained by reaction of PVA with glycidyltrimethylammonium chloride (GTMA-Cl) followed by cross-link formation with glutaraldehyde (GA). The Q-PVA was modified by sol–gel reaction with APTEOS. It was reported that the introduction of ammonium groups enhanced the PVA water permselectivity and permeation flux due to the increased hydrophilicity and reduced PVA crystallinity. Furthermore, the addition of APTEOS introduced nanofractal blisters on the surface due to the self-assembly of the ammonium groups of quaternized PVA chains and the amino groups of APTEOS. The modified membranes showed an improved pervaporation performance with respect to pristine Q-PVA that depended on the APTEOS amount.

**Figure 3:**
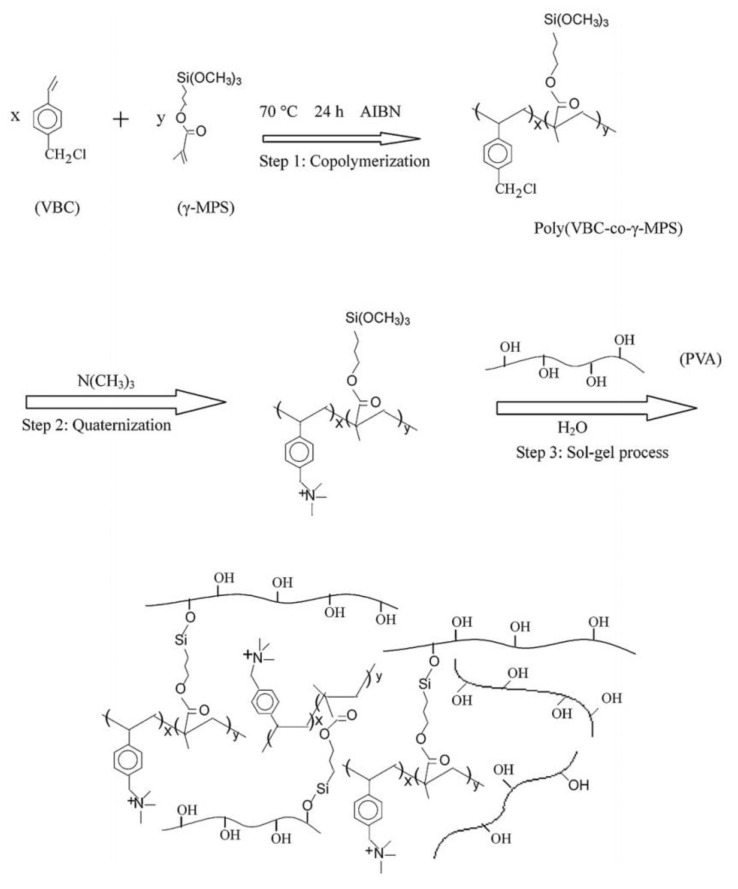
Synthetic routes of hybrid membranes based on poly (VBC-co-MPS) and PVA. Reprinted from Reference [59] with permission from Elsevier.

Co-polymers formed of vinylbenzyl chloride (VBC) and MPS were quaternized and reacted with PVA by the sol–gel process as reported in Figure 3 [59]. They showed excellent properties in DD compared to commercial membranes. The swelling decreased as the amount of co-polymer increased and therefore as the IEC increased, a trend opposite to the normal behavior of AEM resulted because non-functionalized PVA was the main source of membrane swelling.

Poly (VBC-co-MPS) were then prepared with high and low molecular weight and treated with PVA. Here the authors assumed cross-linking of PVA by Si-O-Si bonds and studied the effect of the molecular weight on the membrane properties and DD process [60].

An imidazolium functionalized ionic liquid was directly linked to PVA and TEOS [61]. Membranes showed good DD performance with high acid recovery and separation factor. Imidazolium was also functionalized with APTEOS by sol–gel and used to quaternize BrPPO. The hybrid materials were tested for salt removal [62]. The authors observed that during the process the power consumption decreased while the current efficiency increased with the functionalized silica content.

Another approach was realized preparing 2-(dimethylaminomethyl)pyridine quaternized with a long chain formed by hydroxyl alkylbromide and used as the precursor for sol–gel and cross-linking reactions with PVA and TEOS [63]. A lumped parameter model was developed and used to predict the membrane performance in acid recovery and compared with experimental results.

The double quaternization of 1,4-diazabicyclo [2.2.2] octane (DABCO) was explored in Reference [64] using dibromohexane in the first step and cross-linking with PVA and GPTMOS in the second step. In Reference [65], DABCO was replaced by 1,5-diaminonaphthalene quaternized with GTMA-Cl and reacted with PVA and TEOS that were used as cross-linking agents. The membrane selectivity depended on the ionic radius and mobility and it was very high for Al^3+^ but lower for Fe^2+^ and Zn^2+^.

**Figure 4:**
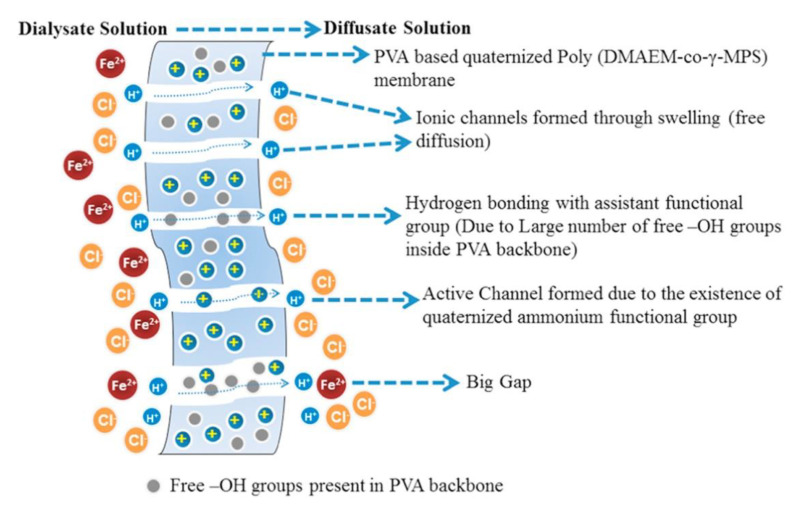
Schematic diffusion dialysis process. Reprinted from Reference [66] with permission from Elsevier.

Poly (DMAEM-co-MPS) was prepared by free radical polymerization of 2-(dimethylaminoethyl) methacrylate (DMAEM) and MPS [66]. The sol–gel precursor was used in the reaction with PVA followed by reaction with GTMA-Cl and quaternization with CH_3_I. A series of membranes was obtained varying the percentage of the co-polymer. In this paper the authors compared the acid recovery and separation performance values of different available membranes for DD and the explanation for the scheme of the process is reported in Figure 4.

Vinod Shahi and his co-workers prepared membranes based on PVA and TEOS by sol–gel and added anion exchange resin particles (Indoin) to the matrix; they studied various membrane properties such as permselectivity and ion transport numbers [67]. In Reference [68] they replaced ion exchange resin particles with quaternized 4-vinylpyridine grafted and were crosslinked to PVA and TEOS. The resulting membranes were analyzed concerning different electrochemical properties, such as ionic transport numbers, electroosmotic coefficient, and ionic conductivity. The conductivity in NaCl was up to 0.4 mS/cm. An anion exchange hybrid material was obtained by a green method using APTEOS and GTMA-Cl via electrophilic ring opening reaction and by introducing the silica precursor in PVA [11]. The presence of the inorganic network in PVA was responsible for good electrochemical properties including a hydroxide conductivity up to 7.6 mS/cm and a low electro-osmotic drag of solvent across the membrane. The same membranes were reported in Reference [69] but the conductivity was much higher.

Free radical polymerization between DMAEM and vinyltrimethoxysilane (VTMS) was followed by sol–gel reaction with PVA, crosslinking, and quaternization [70]. These membranes were analyzed from the point of view of different electrochemical properties including electro-osmotic drag, electrodialysis, and ionic conductivity in NaCl solution (up to 7.2 mS/cm).

Composite membranes for pervaporation separation were developed by Premakshi et al. starting from PVA and a quaternized ammonium silica precursor obtained by APTMOS and GTMA-Cl [71]. The hybrid was obtained by sol–gel reaction and crosslinking with formaldehyde. The increase in the silica content in the membranes increased the permeation flux and the selectivity. The hydrophilicity of the materials favored the selective removal of water from alcohols.

Membranes for acid recovery via DD based on PVA were also obtained by grafting allyltrimethylammonium chloride on the polymer via free-radical polymerization [72]. The grafted polymer was cross-linked with TEOS by sol–gel process. The membrane properties depended on the grafting ratio and an optimal performance (feed solution HCl and FeCl_2_) was obtained with a ratio of 19%, with the best compromise between the permeation flux and the selectivity.

Poly(ethyleneimine) (PEI) was modified by GPTMOS through epoxide ring-opening reaction to synthesized silica modified poly(ethyleneimine) (SMPEI). Composite AEM were obtained by the sol–gel process in aqueous media using PVA [73]. The electro-osmotic study revealed that mass drag across these membranes and their equivalent pore radius increased with SMPEI content in the membrane matrix.

The synthesis of functionalized stationary phases with quaternary ammonium groups on the silica surface was realized by Buszewski et al. [74]. Silica gel was modified with APTEOS and reacted with 4-butanedioldiglycidyl ether and methylamine obtaining quaternized ammonium groups. The resulting materials were efficiently used for the ion-chromatographic separation of inorganic anions.

## 3. Membranes for Electrochemical Energy

Following Reference [44], the hybrid PEO-based matrix was used as the precursor for the subsequent sol–gel reaction with EPh and/or TEOS [75]. The hybrid ionomers intended for use in AEMFC showed homogeneous morphology, good mechanical properties, and relatively good stability in alkali media, although the conductivity was limited and membranes showed water instability.

Similar to the functionalization of PEO, quaternized PPO reacted by sol–gel with EPh and/or TEOS with partially hydrolysed bromomethylated groups and with a supplementary heat treatment at 120–140 °C [76]. According to the authors, a possible effect of the thermal treatment was the formation of cross-linked membranes after loss of the ammonium groups (via Friedel–Crafts reaction) that increased the hydrolytic stability. The conductivity reached 8.5 mS/cm with relatively high alkaline resistance.

In 2008, Xu and his co-workers prepared co-polymers based on VBC and MPS followed by quatenization of VBC with TMA and sol–gel reaction with EPh and/or TEOS [77]. The membranes showed good mechanical properties but the conductivity remained relatively low: 0.2 mS/cm. Other similar membranes obtained with slight modification of the reaction conditions reported in Reference [76] showed apparently better properties including ionic conductivity 8–11 mS/cm at RT and 35 mS/cm at 90 °C in fully humidified conditions [78]. The co-polymer poly (VBC-co-MPS) was blended with BrPPO and then quaternized with TMA [79]. The highest conductivity (12 mS/cm) was observed for a BrPPO content of 75%.

**Figure 5:**
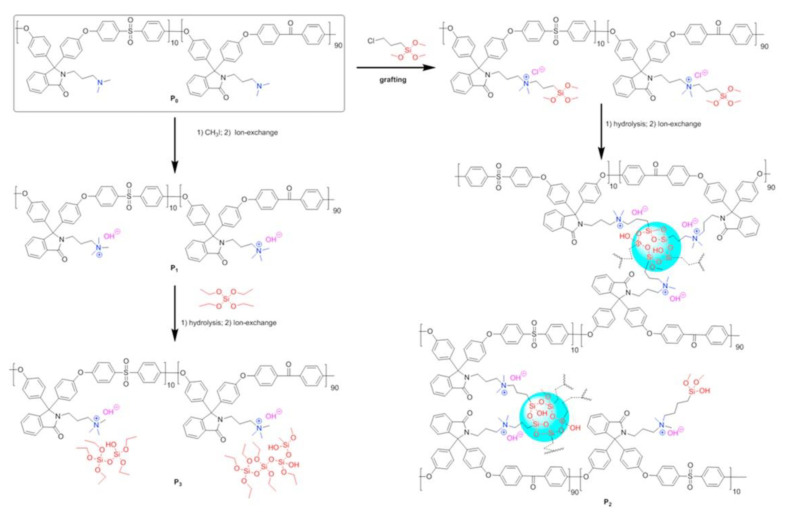
Synthesis routes for composite membrane (**left**) or linked silica network (**right**). Reprinted from Reference [80] with permission from Elsevier.

A comparison between hybrid membranes containing a dispersed or linked silica network was realized by Zheng et al. [80]. Cardo poly (aryl ether sulfone ketone) s functionalized with tertiary amine groups were used as a starting product to prepare composite membranes by in situ sol–gel of TEOS (Figure 5, left). In the second procedure, the same polymer used before quaternization reacted with an alkyl siloxane to obtain a quaternized hybrid precursor. The hybrid polymer then underwent sol–gel reaction via a partial hydrolysis of alkylsiloxane portion in the basic medium. The second procedure led to a hybrid with a Si-O-Si network (Figure 5, right). The membrane with a linked silica network showed an improvement, especially in terms of mechanical properties and alkaline stability, while the composite membranes presented inhomogeneity and poor mechanical resistance.

The synthesis of 4,4′-Oxydiphenylguanidine (ODG) was performed via Vilsmeyer salt and quaternized with (chloromethyl)trimethoxysilane to obtain 4,4′-oxydiphenyl guanidinium-bridged-silsesquioxane (ODGBS) [81]. Chloromethylated polysulfone (PSU) reacted with ODG forming cross-linked and quaternized guanidine moieties and different amounts of ODGBS were added to this system. Ion exchange capacity, water uptake, and conductivity of the modified membrane increased with ODGBS content. The alkaline stability was also related to the ODGBS amount and attributed to better water retention and the delocalization of the positive charge.

**Figure 6:**
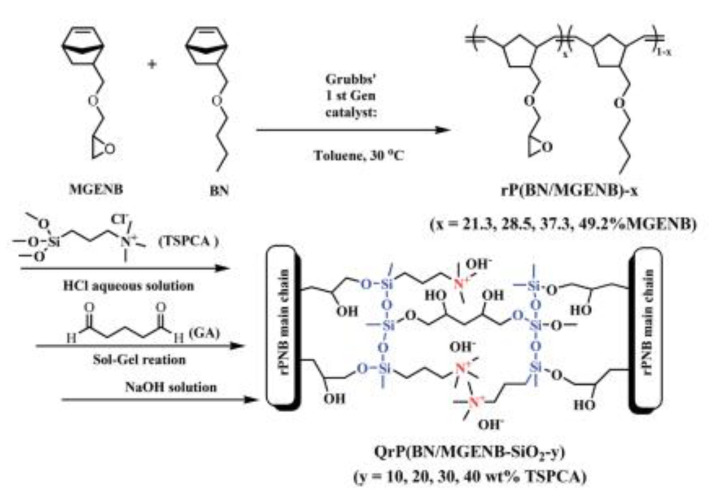
Schematic synthesis of polynorbornene based AEM. Reproduced from Reference [80] with permission from the Royal Society of Chemistry.

ORMOSIL-based membranes can improve another important green technology: Direct Methanol Fuel Cells (DMFC). Low methanol crossover is critical for high energy efficiency and longevity. Membranes based on polynorbornene (PNB) possessing pendant epoxy groups and functionalized by sol–gel with silica-containing ammonium moieties (APTMOS) in the presence of GA as cross-linker were used as separators in DMFC (Figure 6) [82]. The methanol permeability was in the order of 10^−7^ cm^2^ s^−1^, which is much lower than that of Nafion. The alkaline stability in 6M NaOH of the hybrid materials improved with the amount of APTMOS, although the initial conductivity decreased with the presence of the second phase. The best compromise was found for 10% APTMOS. In a modified synthesis, starting from PNB containing hydroxyl groups, the authors investigated the influence of the quantity of quaternary ammonium groups on the properties [83]. The ionic conductivity ascribed to the hydrophilic silica part that was embedded in the PNB hydrophobic matrix reached 10 mS/cm at 80 °C for the sample containing 25% TMSP.

Several AEM were prepared starting from BrPPO. In Reference [84], it was quaternized with 1,2-dimethylimidazole and partially hydrolyzed to react by sol–gel with GPTMOS and TEOS were used in AEMFC. A study of the properties was conducted as a function of the inorganic content and the IEC. The best results were obtained with membranes containing 24% of the inorganic phase and a relatively high value of IEC (2.84 meq/g and conductivity 34 mS/cm at 80 °C). The alkaline stability tested in 2M KOH at 60 °C showed that, for this sample, an improved resistance was obtained (loss of conductivity of pristine and hybrid membranes was 51% and 32% for 10 days, respectively). The dense silica network was believed to be responsible for the higher stability due to its screening effect of the positive charge around the ammonium moiety. A similar beneficial effect was found in composite polyamine (PA) membranes containing a modified silica network [85]. The modified silica, obtained by sol–gel reaction between silica and APTEOS, was cast with PA and the resulting membrane quaternized with CH_3_I.

BrPPO was functionalized by N-methyldiethanolamine and two cross-linker molecules (N,N,N’,N’-tetramethyl-1,6-hexane-diamine and 2-(3,4-epoxycyclohexyl) ethyltrimethoxysilane) were incorporated into the polymeric matrix via sol–gel process. The simultaneous uses of two cross-linking agents were intended to improve the dimensional stability and alkali resistance of the membrane and to maintain a good hydroxide conductivity, which reached 21 mS/cm at 80 °C. The alkaline stability displayed a moderate improvement [86]. Another approach based on BrPPO was exploited by He et al. [87]. The brominated precursor was partially quaternized with triethylamine and reacted with GPTMOS. The epoxy rings reacted with the residual BrPPO while the alkoxy groups of GPTMOS underwent sol–gel reactions forming a cross-linked hybrid material. The conductivity increased with the amount of GPTMOS in the membranes reaching a value of 46 mS/cm at 80 °C. The alkaline stability in 1M KOH at 80 °C showed an improved resistance with respect to the pristine sample although a loss of conductivity around 45% was observed.

Guanidinium-functionalized graphene oxide (GGO) nanoparticles were embedded in un-charged PSU functionalized with diethanolamine (DEA) via the chloromethylation route [88]. The absence of positive charges on PSU was intended to avoid the backbone degradation, especially by the chain scission of ether and sulfone links. The alkaline stability tests in 1M NaOH at 60 °C showed a retention in conductivity around 75% for the sample containing 25% of GGO after 120 h.

Silica particles synthesized by sol–gel were polymerized and quaternized with imidazolium moieties tethered by hydrophilic groups (NH_2_, OH, and CO_2_H) and incorporated in a chitosan CS matrix [89]. The hydrophilicity of the functionalized silica was responsible for good homogeneity and compatibility between the two phases. Despite the relatively low value of IEC (up to 0.45 meq/g) the AEM formed by a non-conductive backbone and conductive particles reached a decent conductivity (up to 3.1 mS/cm at RT). The alkaline stability (3M KOH, 80 °C) was very good; the conductivity loss was less than 10% after 300 h.

**Figure 7:**
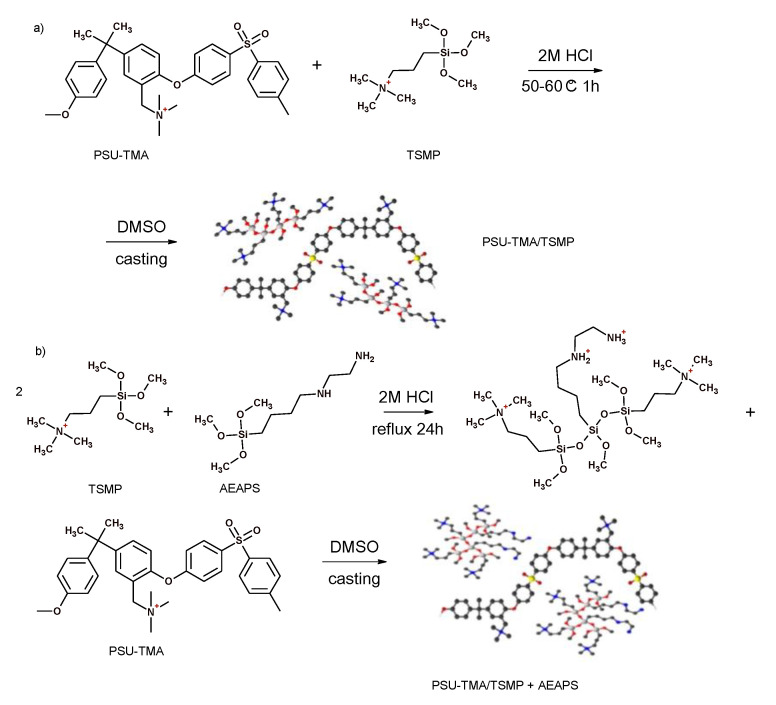
Synthesis routes for hybrid membranes by (**a**) in-situ and (**b**) ex-situ sol–gel techniques [90].

Recently Sgreccia et al. prepared membranes based on TMA-quaternized PSU containing a semi-interpenetrating silica network formed by 3-(trimethoxysilyl) propyl-N,N,N-trimethylammonium chloride (TMSP) or by TMSP and 3-(2-aminoethylamino)propyldimethoxy-methylsilane (AEAPS) [90]. The composite with only TMSP showed better properties in term of ductility and conductivity due to a better homogeneity, although the composite with TMSP and AEAPS presented a more stable network in alkaline conditions (Figure 7).

By adding different amounts of TEOS to PVA quaternized with GTMA-Cl, a series of hybrid membranes with different silica contents were synthesized and used in alkaline DMFC [91]. The membrane with 5 wt% of silica showed the best performances in term of permeability (8.45 × 10^−7^ cm^2^ s^−1^ at 30 °C) and conductivity (6.8 mS/cm). A pyridine-functionalized PVA matrix was also prepared and the hydroxy groups of PVA reacted with ethoxy groups of 3-(2-aminoethylamino)propyltriethoxysilane (AAPTEOS) to form a network of Si-O-C and Si-O-Si bonds, which improved the dimensional stability and mechanical properties of the hybrid membranes [92]. The highest conductivity was 14 mS/cm at 30 °C and 96 mS/cm at 80 °C and the alkaline stability was remarkable; after 360 h in 6M NaOH at 80 °C, the remaining conductivity was nearly 90% of the initial one.

For vanadium redox flow batteries, Zhao et al. [93] prepared hybrid AEM starting from commercial Fumasep FAP (fluorinated AEM, Fumatech GmbH, Bietigheim-Bissingen, Germany), which was reacted by in situ sol–gel reaction with TEOS. The effects of using silica nanoparticles were the reductions in the crossover of vanadium ions and the rates of self-discharge and capacity loss. Hybrid membranes were also prepared by reaction of (CS) and GPTMOS followed by sol–gel process [94]. These membranes did not contain permanent ammonium groups but became AEM by reaction of amine groups with an acid solution. The IEC remained relatively low (<0.7 meq/g) and the conductivity in 1 M VOSO_4_ and 1M H_2_SO_4_ was in the order of 10 mS/cm.

## 4. Electrodialysis (ED)

Organic–inorganic AEM were synthetized to evaluate the performance of anion exchangers in the NaCl removal from an aqueous solution [95]. PVA, TMA-quaternized CS, and an anion-exchange silica precursor formed by an epoxide ring opening reaction between glycidoxypropyltrimethylammonium chloride and AAPTMOS were reacted and crosslinked by sol–gel in acidic conditions. The energy consumption was measured (4.1 kWh/kg for 94% removal of NaCl from 0.2 M NaCl).

Water-soluble siloxane resins obtained by sol–gel reaction of N-dimethoxymethylsilylpropyl-N,N,N-trimethylammonium chloride and 3-acrylamido- propyltrimethoxysilane were blended with monomers containing ammonium and acrylamide moieties and further submitted to photoinduced radical polymerization [96]. The obtained blend was also used for the impregnation of porous polyethylene to synthetize pore-filling hybrid AEM. The properties of the samples were influenced by the resin composition. The membranes were used for reverse ED showing an improved performance with respect to commercial AEM.

Graphene nano-ribbons bearing amide groups (fGNR) were incorporated into a co-polymer formed by free radical copolymerization between VBC quaternized with N-methylmorpholine and triethoxyvinylsilane. The co-polymer underwent sol–gel reaction with PVA and was cross-linked with formaldehyde [97]. The hydroxide ion conductivity reached 12 mS/cm at RT for the higher amount of fGNR (0.1 wt%). The hybrid membranes were used for salt removal by ED and for acid recovery by DD. In the ED process, the composite with the higher amount of fGNR showed the best current efficiency (89%) and the best lower energy consumption (1.36 kWh/kg).

**Figure 8:**
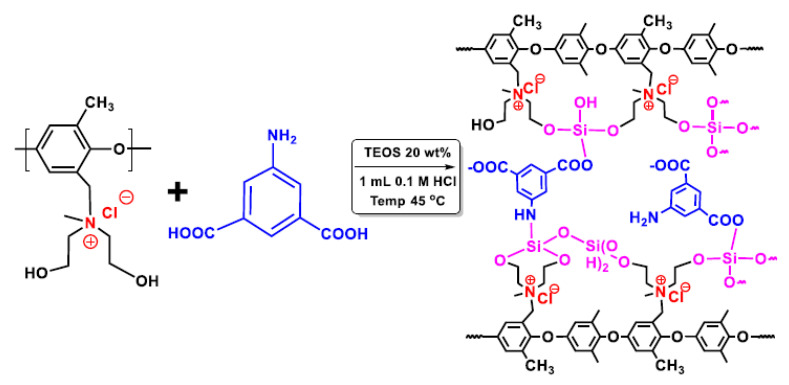
Zwitterionic membrane for electrodialysis. Reprinted from Reference [98] with permission from Elsevier.

Xu and his co-workers prepared zwitterionic membranes by the introduction of carboxylic acid groups in the membrane matrix via a sol–gel process with PPO quaternized with DEA (Figure 8). They investigated the influence of the amino isophthalic acid (AIPA) content on the membrane ED properties, such as ion flux and permselectivity for the Li^+^/Mg^2+^ system, as a model for the separation of lithium from seawater. The membrane with 20 wt% AIPA showed high flux with good permselectivity. Furthermore, excellent flux and permselectivity were also found for the other ions, including Na^+^/Mg^2+^, K^+^/Mg^2+^, and H^+^/Fe^2+^ [98].

This section may be divided by subheadings. It should provide a concise and precise description of the experimental results, their interpretation, as well as the experimental conclusions that can be drawn.

Another zwitterionic polymer was prepared by reaction of AEAPS and 3-cyanopropyltrichlorosilane with PSU. This polymer showed pH sensitive properties, such as tuneable ionic conductivity and interface potential deposition on acidic and basic substrates [99].

## 5. Salient Features

**Figure 9:**
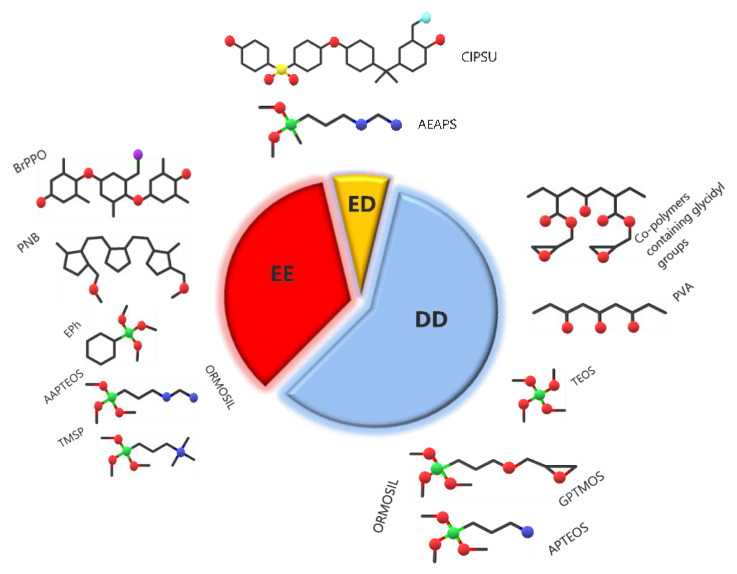
Relative amount of papers on the three main applications of sol–gel AEM and major polymer precursors and Si sources for these applications.

Figure 9 represents the share of works on the three main applications of sol–gel AEM; the largest part is devoted to diffusion dialysis, followed by electrochemical energy technologies and electrodialysis. The most popular precursor polymers and Ormosils are also schematized in the figure.

There are little literature on sol–gel modified AEM with other elements than Si [100,101]. According to many authors, the SiOH groups in sol–gel silica interact strongly with water molecules and favor water retention. Excessive condensation into Si-O-Si network can, however, limit the formation of ionic channels and much effort is directed to the correct the balance of the two phases. Titania and zirconia precursors, due to the higher reactivity of the metal, generally lead to a more condensed network with less OH groups. Furthermore, the starting materials for transition metal sol–gel methods are not easily available, more expensive, and sometimes difficult to manage.

Table 1 summarizes the precursors and the important properties of sol–gel composite AEM. One can observe that the ionic conductivity at room temperature remains below 10 mS/cm in most cases, although few papers indicate surprisingly high values. Similarly, the proton dialysis coefficients and separation factors are mostly consistent with few exceptions. In electrochemical energy technologies, the composite AEM are mainly used in DMFC, because the second phase can further reduce the methanol permeability.

By far, the largest amount of papers is devoted to the development of AEM for DD and related processes, especially with membranes including a PVA matrix. Glycidyl groups in the polymer backbone are often used to link the organo-silica network. AEM presents several advantages for acid recovery, especially high product quality given the high selectivity for acids [102]. An important requisite for the matrix is its hydrophilicity that helps the water diffusion; for this reason, PEO and PVA are very popular. However, a very hydrophilic matrix may lead to high swelling and poor mechanical properties, which can be counterbalanced by the incorporation of cross-linking agents or an organo-silica network.

In electrochemical technologies and electrodialysis, the choice of membranes is more diverse because ionic conductivity and membrane stability in alkaline conditions play a central role. The largest amount of work was made on PPO-based systems. The improvement regarding the stability is not always evident. Some attempts to achieve AEM without a charged organic polymer backbone were made. In many cases, the positive ionic groups are grafted onto the organic polymer, which can degrade the alkaline stability due to backbone scission reactions, especially if the polymer backbone contains ether groups. The possibility to anchor the positive groups to the organo-silica networks might improve the alkaline stability of the composite AEM. However, a clear experimental confirmation remains to be produced.

## 6. Conclusions

This short article reviews the field of composite AEM, including a hybrid organic–inorganic second phase built in-situ by sol–gel chemistry. The main applications covered are AEM for diffusion dialysis, especially acid recovery; electrochemical energy technologies, including AEM for fuel cells and redox flow batteries; and AEM for electrodialysis.

The DD process performance is linked to the diffusion flux and the membrane hydrophilicity and permselectivity. The permselectivity depends on the characteristics of the ions (size, charge, and mobility), the morphology, and the composition of IEM. Composites based on PVA are the most developed, while simultaneously placing at a lower price. The silica part improves the weak mechanical properties of pristine PVA.

Another main requirement for fuel cells, redox flow batteries, and electrodialysis is a high ionic conductivity; furthermore, a good alkaline stability is necessary for AEMFC. Composites based on many different polymers were reported with the largest share for PPO-based AEM. The improvement of alkaline stability by the addition of a silica network remains to be confirmed. In our opinion, the alkaline stability might be further improved for AEM when the ionophoric groups are placed at the organo-silica part which may reduce the organic polymer chain scissions, especially at ether groups.

There is also a variety of polymer matrices used in ED. An interesting approach for ED is the development of zwitterionic membranes that can conduct both cations and anions.

## Figures and Tables

**Table 1:** Polymer precursors, Si sources, and important properties of sol–gel modified AEM.

Polymer Matrix	Si Precursor	Properties	Reference
PEO	N-[3-(trimethoxysilyl)propyl] ethylene diamine	Pore size decreases with the increase in dip-coating sols (0.2–0.6, 0.023–0.12, 0.008–0.033 and 0.002–0.006 μm)	[44]
Poly(GMA-co-MPS)	MPS	Pore diameters: 0.006–0.002 μm	[45]
Poly(GMA-co-MPS)	MPS APTEOS-I, EPh	APTEOS-I content controls the membrane electrical properties Membrane potential: 11.6–15.8 mV	[47]
Poly(MPS-co-GPTMOS)	GPTMOS TEOS; Triethoxysilylpropylammonium	Membrane potential: 14.8–18.8 mV Transport number > 0.92	[48]
BrPPO	APTMOS	Mole ratio Si/PPO in the polymer precursor 0.34, 0.42, 0.62, and 0.91	[49]
PPO-TMA hollow-fibers	TEOS	Maximum water uptake: 1.4 g water/g dry hollow fiber (10% of TEOS) Dimensional change: 13–16%	[50]
PPO-TMA	EPh TEOS	Easy diffusion of H^+^ and Fe^2+^ (45 °C) at high content of silica: molar ratio (TEOS + EPh)/Br-PPO = 32%	[51]
PPO	SiO_2_	Proton dialysis coefficient: 0.0703 m/h Separation factor (HCl/FeCl_2_): 68.0	[52]
PPO-DMAE	IL: 3-methyl-1-(3-(triethoxysilyl)propyl)-1H-imidazolium chloride	Proton dialysis coefficient: 0.020–0.0273 m/h Separation factor (HCl/FeCl_2_): 38.8–86.5	[53]
PPO-DMAE	IL: 3-methyl-1-(3-(triethoxysilyl)propyl)-1H-imidazol-3-ium chloride	Zwitterionic pores Proton dialysis coefficient: 0.021–0.0386 m/h Separation factor (HCl/FeCl_2_): 33.9–62.0	[54]
PVA	APTEOS-I TEOS, GPTMOS, EPh; GMA-MPS	Separation factor (HCl/FeCl_2_): 16–21	[55]
PPO-TMA PVA	TEOS EPh	Proton dialysis coefficient: 0.021–0.049 m/h Separation factor (HCl/FeCl_2_): 44	[56]
PPO-TMA PVA	TEOS EPh	Proton dialysis coefficient: 0.008–0.011 m/h (15 °C), 0.014–0.018 m/h (55 °C) Separation factor (HCl/FeCl_2_): 48–68 (15 °C), 40–51 (55 °C)	[57]
Poly(PVA-co-GTMA-Cl)	APTEOS	Separation factor (85% ethanol in water): 52–63 (50 °C) Nanofractal blisters on the surface	[58]
Poly(VBC-co-MPS) PVA	MPS	Separation factor (HCl/FeCl_2_): 25–30 (20 °C), 12.1–35.7 (60 °C)	[59]
Poly(VBC-co-MPS) (high and low molecular weight) PVA	MPS	Proton dialysis coefficient (CH_3_COOH): 0.009 m/h; Proton Dialysis coefficient (HCl): 0.01–0.029 m/h) Separation factor (HCl/FeCl_2_): 28–39	[60]
PVA	TEOS 1-methylimidazole-AESP	Proton dialysis coefficient 0.0315–0.0483 m/h Separation factor (HCl/FeCl_2_): 28.6–52.5	[61]
PPO-triethylamine	1-vinylimidazole-APTEOS	Power consumption: 0.98–1.17 kWh/Kg Current efficiency: 74.02–89.73%	[62]
PVA-2-(dimethylaminomethyl)pyridine	TEOS	Proton dialysis coefficient: 0.009–0.022 m/h Fe^2+^ dialysis coefficient: 0.00017–0.00055 m/h Separation factor (HCl/FeCl_2_): 42–54	[63]
PVA-DABCO	GPTMOS	Proton dialysis coefficient: 0.03–0.045 m/h Fe^2+^ dialysis coefficient: 0.0009–0.0015 m/h Separation factor (HCl/FeCl_2_): 20.9–32.3	[64]
PVA-1,5-diaminonaphthalene-GTMA-Cl	TEOS	Proton dialysis coefficient: 0.0225 m/h (HCl-NaCl); 0.025 m/h (HCl-ZnCl_2_); 0.0275 m/h (HCl-FeCl_2_); 0.026 m/h (HCl-AlCl_3_)	[65]
Poly(DMAEM-co-MPS) PVA	MPS	Proton dialysis coefficient: 0.016–0.029 m/h Separation factor (HCl/FeCl_2_): 23.3–87.7	[66]
PVA Anion exchange resin particles (Indoin)	TEOS	Counter-ion transport numbers: 0.910–0.916 (Cl^−^); 0.785–0.838 (Br^−^); 0.712–0.786 (F^−^) Permselectivity: 0.775–0.790 (Cl^−^); 0.462–0.594 (Br^−^); 0.387–0.545 (F^−^)	[67]
PVA-4-vinylpyridine	TEOS	Electroosmotic permeability: 0.41–2.17 × 10^−4^ cm^3^/C Counterion transport number: 0.92	[68]
PVA GTMA-Cl	APTEOS	Counter ion transport number: 0.91–0.96 Permselectivity (OH^−^): 0.86–0.94 OH^−^ ion conductivity: 5.9–7.6 mS/cm	[11]
PVA GTMA-Cl	APTEOS	Transport number: 0.79–0.92 OH^−^ ion conductivity: 34.8–75.7 mS/cm	[69]
DMAEM-VTMS PVA	VTMS	Permselectivity (Cl^−^): 0.76–0.90 Cl^−^ ion conductivity: 7.2 mS/cm	[70]
PVA	APTEOS -GTMA-Cl	Tensile strength: 55–69 MPa Elongation at break: 45–80% IEC: 0.25–0.73 meq/g	[71]
PVA-allyltrimethylammonium chloride	TEOS	Proton dialysis coefficient: 0.015–0.060 m/h Fe^2+^ dialysis coefficient: 0.013–0.020 m/h Separation factor (HCl/FeCl_2_): 7–22	[72]
SMPEI, PVA	GPTMOS	Permselectivity (Cl^−^): 0.79	[73]
4-butanedioldiglycidyl ether-methylamine	Silica Gel APTEOS	Retention factor: 2.08–4.10 (Cl^−^); 3.59–7.01 (Br^−^); 1.02–1.88 (F^−^); 4.42–8.57 (NO_3_^−^); 2.10–5.64 (NO_2_^−^)	[74]
PEO	EPh TEOS	Tensile Strength: 1.0–20.5 MPa Elongation at break: 33–120% OH^−^ ion conductivity: 3 mS/cm	[75]
PPO-triethylamine	EPh TEOS	IEC (Br^−^ form) 1.27–2.05 mmol/g OH^−^ ion conductivity: 1.0–8.5 mS/cm	[76]
Poly(VBC-co-MPS)	MPS EPh, TEOS	IEC (Cl^−^ form) 1.70–2.20 mmol/g OH^−^ ion conductivity: 0.2–0.4 mS/cm	[77]
PPO-triethylamine	EPh TEOS	OH- ion conductivity: 8–11 mS/cm (RT); 35 mS/cm (90 °C) Peak power density: 32 mW/cm (fuel cell test)	[78]
Poly(VBC-co-MPS) BrPPO	MPS	IEC (Cl^−^ form): 2.20–2.25 mmol/g OH^−^ ion conductivity: 12 mS/cm	[79]
Cardo poly(aryl ether sulfone ketone	3-Chloropropyltrimethoxysilane TEOS	Tensile Strength: 20.0–40.3 MPa Young’s Modulus: 196–1166 MPa Elongation at break: 36–70%	[80]
PSU	ODGBS	OH^−^ ion conductivity: 20–26 mS/cm (60 °C)	[81]
PNB	APTMOS	Methanol permeability: 1.54–2.75 × 10^−7^ cm^2^/s OH^−^ ion conductivity 6.3–41.0 mS/cm Peak power density: 43 mW/cm (fuel cell test)	[82]
PNB	TMSP	Methanol permeability: 1.34–2.89 × 10^−7^ cm^2^/s OH^−^ ion conductivity (80 °C): 6.8–9.3 mS/cm Peak power density: 32 mW/cm^2^_;_(fuel cell test)	[83]
PPO-1,2-dimethylimidazole	GPTMOS TEOS	IEC: 2.19–2.63 mmol/g OH^−^ ion conductivity: 10–22 mS/cm (25 °C); 26–36 mS/cm (80 °C)	[84]
5PA	silica and APTEOS	IEC: 1.29 mmol/g	[85]
PPO-N-methyldiethanolamine	2-(3,4-epoxycyclohexyl) ethyltrimethoxysilane	OH^−^ ion conductivity: 21 mS/cm Peak power density: 14.2 mW/cm^2^ (40 °C); 16.9 mW/cm^2^ (60 °C) (single cell test)	[86]
PPO-triethylamine	GPTMOS	OH^−^ ion conductivity: 46.0 mS/cm (80 °C)	[87]
PSU-DEA GGO	APTMOS	IEC: 0.48–0.90 mmol/g OH^−^ ion conductivity: 6–11 mS/cm (RT); 12–20 mS/cm (70 °C)	[88]
CS	3-(Methacryloxy) propyl-trimethoxysilane TEOS	IEC: 0.37–0.46 mmol/g OH^−^ ion conductivity: 1–3 mS/cm (20 °C); 6–13 mS/cm (90 °C)	[89]
PSU-TMA	TMSP AEAPS	IEC: 1.3–1.4 mmol/g Cl^−^ ion conductivity: 0.8–1.3 mS/cm (RT); 3.4–3.9 mS/cm (80 °C)	[90]
PVA- GTMA-Cl	TEOS	Methanol permeability: 8.4–11.6 × 10^−7^ cm^2^/s. OH^−^ ion conductivity: 3.1–6.8 mS/cm (30 °C); 14 mS/cm (60 °C)	[91]
PVA- pyridine	AAPTEOS	Peak power density: 53 mW/cm^2^ (80 °C) (fuel cell test) Alkaline stability: remaining conductivity 90% (360 h in 6M NaOH at 80 °C)	[92]
Fumasep FAP	TEOS	IEC: 1.07–1.13 mmol/g VO^2+^ permeability: 5.48 × 10^−7^ cm^2^/min	[93]
CS	GPTMOS	IEC: 0.34–0.71 mmol/g VO^2+^ permeability: 3.13–8.17 × 10^−6^ cm^2^/min SO_4_^2-^ ion conductivity: 7.6–11.3 mS/cm	[94]
PVA CS-TMA	AAPTMS TEOS	IEC: 0.82–1.29 mmol/g Chloride ion transport number: 0.86–0.94	[95]
(3-acrylamidopropyl)-trimethylammonium, Polyethylene	Siloxane resins	IEC: 1.67–2.26 mmol/g Resistance: 0.23–0.32 Ω/cm^2^	[96]
poly(QVBC-co-triethoxyvinylsilane) PVA, Graphene nano-ribbons	Triethoxyvinylsilane	IEC: 1.92–2.09 meq/g Energy consumption: 1.36 kWh/kg	[97]
PPO-DEA	AIPA	Zwitterionic membrane Permselectivity: 8 (Li^+^/Mg^2+^); 24.8 (K^+^/Mg^2+^); 41.3 (Na^+^/Mg^2+^); 261.7 (H^+^/Fe^2+^)	[98]
PSU	AEAPS 3-cyanopropyltrichlorosilane	Zwitterionic membrane 0.071 mS/cm (acidic), 0.051 mS/cm (basic), 0.0065–0.0088 mS/cm (zwitterionic) (80 °C)	[99]

## Data Availability

Not applicable.

## Abbreviations

AAPTEOS	3-(2-aminoethylamino)propyltriethoxysilane
AAPTMOS	3-(2-aminoethylamino)propyltrimethoxysilane
AEAPS	3-(2-aminoethylamino)propyldimethoxymethylsilane
AEM:	anion exchange membranes
AEMFC	anion exchange membrane fuel cells
AIPA	amino-isophthalic acid
APTEOS	3-aminopropyltriethoxysilane
APTMOS	3-aminopropyltrimethoxysilane
APTEOS-I	N-triethoxysilylpropyl-N,N,N-trimethylammonium iodide
BrPPO	brominated poly(2,6-dimethyl-1,4-phenylene oxide)
CS	chitosan
DABCO	1,4-diazabicyclo[2.2.2]octane
DD	diffusion dialysis
DEA	diethanolamine
DMAE	dimethylaminoethanol
DMAEM	2-(dimethylaminoethyl) methacrylate
ED	electrodialysis
EPh	monophenyltriethoxysilane
GA	glutaraldehyde
GMA	glycidylmethacrylate
GPTMOS	3-glycidoxypropyltrimethoxysilane
GTMA-Cl	glycidyltrimethylammonium chloride
MPS	3-methacryloxypropyltrimethoxysilane
ODG	4,4′-oxydiphenylguanidine
PA	polyamine
PEI	poly(ethylene imine)
PEO	poly(ethylene oxide)b
PNB	polynorbornene
PPO	poly(2,6-dimethyl-1,4-phenylene oxide)
PSU	polysulfone
PVA	poly(vinyl alcohol)
QPVA	quaternized poly(vinyl alcohol)
SMPEI	silica-modified poly(ethylene imine)
TEOS	tetraethoxysilane
TMA	trimethylamine/trimethylammonium
TMSP	N-trimethoxysilylpropyl-N,N,N-trimethylammonium chloride
VBC	vinylbenzyl chloride
VTMS	vinyltrimethoxysilane
